# Genomic prediction for testes weight of the tiger pufferfish, *Takifugu rubripes*, using medium to low density SNPs

**DOI:** 10.1038/s41598-021-99829-1

**Published:** 2021-10-13

**Authors:** Sho Hosoya, Sota Yoshikawa, Mana Sato, Kiyoshi Kikuchi

**Affiliations:** 1 Fisheries Laboratory, University of Tokyo, Hamamatsu, 431-0214 Japan; 2 Nagasaki Prefectural Institute of Fisheries, Nagasaki, Japan

**Keywords:** Animal breeding, Heritable quantitative trait, Genetic markers

## Abstract

Aquaculture production is expected to increase with the help of genomic selection (GS). The possibility of performing GS using only a small number of SNPs has been examined in order to reduce genotyping costs; however, the practicality of this approach is still unclear. Here, we tested whether the effects of reducing the number of SNPs impaired the prediction accuracy of GS for standard length, body weight, and testes weight in the tiger pufferfish (*Takifugu rubripes*). High values for predictive ability (0.563–0.606) were obtained with 4000 SNPs for all traits under a genomic best linear unbiased predictor (GBLUP) model. These values were still within an acceptable range with 1200 SNPs (0.554–0.588). However, predictive abilities and prediction accuracies deteriorated using less than 1200 SNPs largely due to the reduced power in accurately estimating the genetic relationship among individuals; family structure could still be resolved with as few as 400 SNPs. This suggests that the SNPs informative for estimation of genetic relatedness among individuals differ from those for inference of family structure, and that non-random SNP selection based on the effects on family structure (e.g., site-*F*_*ST*_, principal components, or random forest) is unlikely to increase the prediction accuracy for these traits.

## Introduction

Aquaculture as an industry has taken root worldwide and has become the fastest growing in the food production sector^1^. However, most cultured species are genetically still very similar to their wild conspecifics because of their relatively short history under culture. Therefore, significant genetic variation exists in cultured populations that can be harnessed to boost aquaculture production by means of selective breeding^2^. For example, an average of 13% of genetic gain in growth rate per generation has been achieved in salmonids^3^. Such genetic improvements can be further accelerated by incorporating recent developments in molecular tools, such as genomic selection (GS)^4^.

The theory of GS was first proposed by Meuwissen et al.^5^ and is now implemented in many aquaculture species^4^. In this approach, a prediction model is trained by regressing the phenotype on the genome-wide genotype of a reference population, and genomic estimated breeding values (GEBVs) of selection candidates are predicted by substituting their genotypes in the model (termed genomic prediction, GP). Genomic selection is particularly advantageous for aquaculture species because many of economically important traits are polygenic (e.g., growth and disease resistance), and cultured populations often consist of many full-sib/half-sib families resulting in highly accurate GP^6^. Among GP models, the genomic best linear unbiased predictor (GBLUP) model is widely used for polygenic traits^7,8^. GBLUP is statistically very similar to the traditional pedigree-based BLUP (PBLUP); the main difference is that the pedigree-based relationships used in PBLUP are replaced by genomic relationships inferred from genome-wide SNP information. Overall GBLUP often outperforms PBLUP in prediction accuracy because genomic relationships can estimate the fraction of a genome shared between individuals more accurately than pedigree-based relationships; the latter are based on expected values while the former evaluate deviations around the expected values (i.e., Mendelian sampling)^9^. The major drawback of GS is that frequent genotyping for thousands of SNPs in hundreds of individuals is required for accurate prediction. Therefore, extensive efforts are underway to reduce genotyping costs, such as studying the effect of sib-test frequency on prediction accuracy^10^, using genotype-by-sequencing methods^11^, and using low-density markers with imputation^12,13^. It is expected that a relatively small number of SNPs might be sufficient to perform GP in aquaculture because breeding populations often include many full- and half-sib families, and the genomic relationships among population members can be accurately captured with a few thousand SNPs. Indeed, recent studies have suggested that near-maximal prediction accuracy can be gained with a low-density SNP panel (1000 SNPs) regardless of the differences in population and family structure, phenotype, and trait definition^14^. However, the practical utility of GS with a small number of SNPs is still unclear and needs to be tested with more species and traits.

In this study, we examined the feasibility of GP for testes weight (TW) in the tiger pufferfish (*Takifugu rubripes*) and sought to determine the minimum number of SNPs required to achieve good prediction accuracy for TW, standard length (SL), and body weight (BW). The tiger pufferfish (also known as fugu, a genomic model fish^15,16^) is one of the most valuable aquaculture species in Japan^17^. Artificially raised seedlings have been used in aquaculture of this species since the 1990s when technologies for broodstock management and artificial seed production were established^18–21^. Aquaculture production using these methods now dominates (approximately 90%) overall production of this species (Japan Fisheries Research and Education Agency (FRA), Japan: http://abchan.fra.go.jp/digests2019/index.html, accessed 12 Nov 2020). Although a selective breeding program for this species is still in its infancy^17^, the possibility of using GS for SL, BW, and for resistance against the monogenean parasite, *Heterobothrium okamotoi*, has already been tested^22^. Precociousness is another important economic trait in this species^23^. While the ovary is highly poisonous, the testis is edible and is the most expensive of the edible parts^24^. In most individuals, the testes reach marketable size (> 100 g) in late January or February (when the fish is about 22 months old); however, some individuals have testes larger than 100 g in early December (at about 20 months old), a time when prices are also at their highest. As precociousness is a polygenic trait^23,25^ then TW at harvest can potentially be improved by GS. To test this possibility, we raised an experimental population and applied GP for TW, SL, and BW using a genome-wide medium density SNP panel. Pedigree information was reconstructed using genomic information to enable application of the BLUP method. We also examined the effect of reducing the number of SNPs (from 4075 to 200) on the prediction accuracy and estimation of genetic relatedness among individuals to test the feasibility of using a low-density SNP panel for GS.

## Results

### Phenotypic distribution

**Figure 1:**
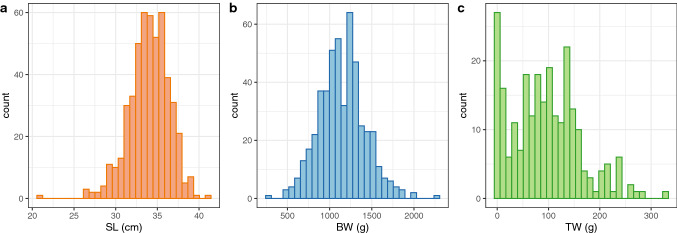
Distribution of standard length (SL) (**a**), body weight (BW) (**b**), and testes weight (**c**) of F_1_ individuals at harvest.

The test generation (F_1_), consisting of 129 full-sib families, was produced from nine females and nineteen males of the founder generation (F_0_) in April 2018. The parental individuals were obtained from 996 fish purchased from aquaculture farms in Nagasaki prefecture (Japan) in December 2016. Of these, 652 were used in the previous study^26^ and the remaining 344 were utilized in this study. The F_1_ generation included 712 individuals; these were at 20 months old and fin-clips were collected. Body size data (SL and BW) were collected from 695 individuals while TW was obtained from the 246 males. The phenotypic data (SL, BW, and TW) of the F_1_ specimens is shown in Fig. 1.

### Pedigree reconstruction

As the genetic origin of each parent and the parental pair of each individual were unknown, pedigree information was reconstructed using 4075 genome-wide SNPs obtained using the Ampliseq custom panel (Thermo Fisher Scientific Inc). The results of the kin relationship analysis among parents and the parent–offspring assignments were combined to reconstruct pedigree information of the population. Based on this inferred pedigree, a numerator relationship matrix (A matrix) was constructed for further genetic analyses.

Kin relationship among parental individuals was inferred using KING software^27^; with the exception of one female parent, the parental individuals comprised six full-sib families and two pairs of half-sibs. The exceptional female parent was estimated to be a third-degree relative of one pair of half-sibs (Supplementary Fig. S1).

**Figure 2:**
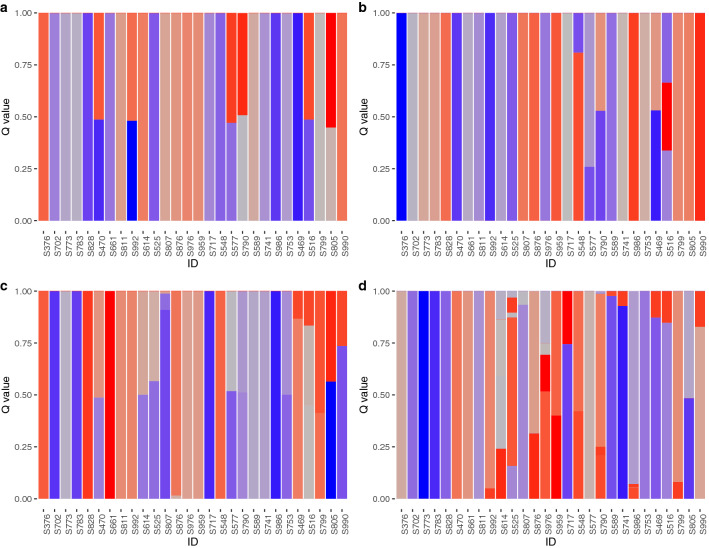
Genetic admixture in families identified using four different SNP datasets: (**a**) 4075 SNPs; (**b**) 800 SNPs; (**c**) 400 SNPs; (**d**) 200 SNPs. Only the results of parental individuals are shown here. The results of siblings are summarized in Supplementary Table S1. Vertical columns indicate estimated admixture proportions of each parental fish. Color expressed in the Kelvin scale represent each of the *K* axes.

Parentage assignments were carried out by means of genetic admixture analysis^28^ using the full SNP dataset and also three subsets of imputed SNPs (800, 400, and 200 SNPs) to examine the effect of the number of SNPs for the assignments. We selected the optimal *K* value for each SNP set based on a five-fold cross validation: *K* = 34 for the full SNP data set, and *K* = 33, 38, and 34 for the 800, 400, and 200 SNPs subsets, respectively (Supplementary Fig. S2). The same family structure was captured among the full SNP dataset and 800 and 400 SNPs subsets; every parental individual was clearly distinguished, each sibling was uniquely assigned to a single parental pair, and the parent–offspring pairing results were congruent among the three data sets (Fig. 2 and Supplementary Table S1). In total, 129 full-sib families were identified, and this number matched with the crossing record (Supplementary Table S2), supporting the accuracy of pedigree reconstruction and indicating that at least one individual was sampled from each full-sib family. On the other hand, we were unable to assign some siblings to a single parental pair with the 200 SNPs subset, as multiple individuals shared the same *K* axes.

### Heritability estimation and breeding value prediction

**Figure 3:**
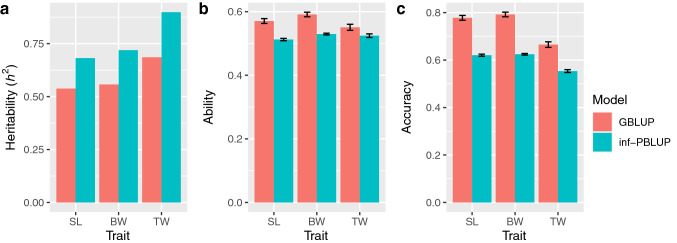
Estimates of (**a**) narrow sense heritability (*h*^2^), (**b**) predictive ability, and (**c**) prediction accuracy for standard length (SL), body weight (BW), and testes weight (TW) using the genomic best linear unbiassed predictor (GBLUP) and inferred pedigree based BLUP (inf-PBLUP) models. Bars indicate standard error of the mean for ten replicates.

To test the feasibility of GS for TW in the tiger pufferfish, the narrow sense heritability (*h*^2^), predictive ability, and prediction accuracy were estimated along with those for SL and BW under GBLUP using the full SNP data (4075 SNPs) (Fig. 3 and Supplementary Table S3). These values were compared with those estimated under the inferred pedigree BLUP (inf-PBLUP) model.

The estimated heritability under the GBLUP model was moderate for every trait, with that for TW being the highest (TW: *h*^2^ = 0.686; SL: 0.538; BW: 0.557), these values were smaller than those estimated under the inf-PBLUP model (TW: 0.899; SL: 0.682; BW: 0.719). The predictive ability and prediction accuracy were also high for each trait under the GBLUP model. The predictive ability (measured as the correlation coefficient between GEBV and observed phenotypes) were similar among the three traits (0.551–0.591), while the prediction accuracy (the prediction ability divided by square-root of the heritability) varied somewhat (0.665–0.792). Both values were highest for BW and lowest for TW. These values were higher than those estimated under the inf-PBLUP model (ability: 0.515–0.532; accuracy: 0.544–0.627).

### Effect of reduced number of SNPs on prediction

**Figure 4:**
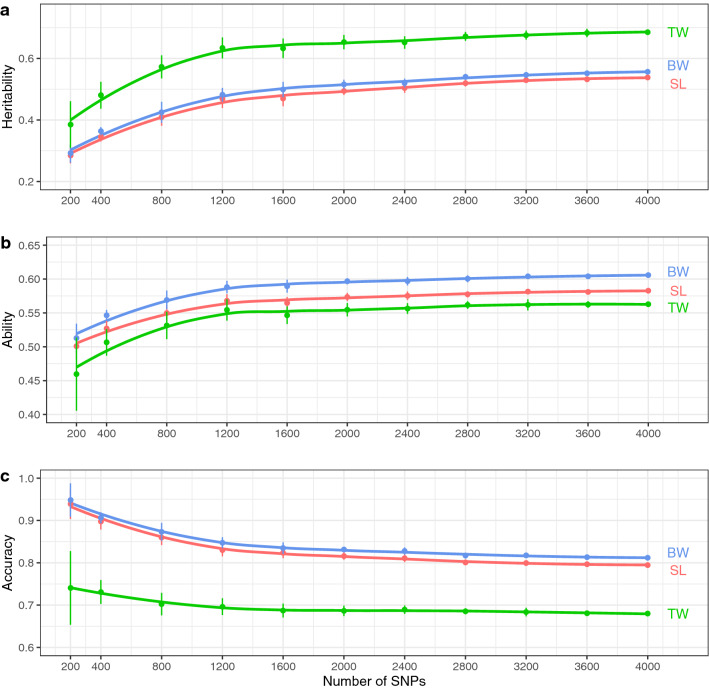
Effect of reducing the number of SNPs on prediction. Heritability (*h*^*2*^) (**a**), predictive ability (**b**), and prediction accuracy (**c**) for standard length (SL), body weight (BW), and testes weight (TW) were calculated using subsets with different numbers of SNPs under the GBLUP model. Each SNP subset was sampled with ten replicates, and the mean and the standard deviation among the replicates are plotted. Loess regression lines (span = 0.75) are shown.

To examine the effect of a reduction in the number of SNPs on GP, we extracted random subsets with different numbers of SNPs (200, 400, 800, 1200, 1600, 2000, 2400, 2800, 3200, 3600, and 4000) from the full SNP set (4075 SNPs in total). For each subset, SNP extraction was performed independently ten times (i.e., ten replicates per subset). Unsurprisingly, the ten replicates of the subset with 4000 SNPs were nearly identical to each other as these were selected from 4075 SNPs. Genetic parameters were estimated under the GBLUP model using these subsets and compared with the results obtained using the full SNP data sets (Fig. 4).

Irrespective of the trait studied, the heritability value decreased slowly until about 1200 SNPs and fell rapidly thereafter as the number of SNPs decreased (Fig. 4a). Compared to the full SNP set, a 16–25% reduction was seen in the heritability using 800 SNPs and an approximately 40% reduction with 200 SNPs. Similarly, a drop in predictive ability was observed when the number of SNPs were fell below 1200 for each trait (Fig. 4b). In contrast, prediction accuracy was inflated with small SNP subsets, due to the rapid decrease in the heritability, since accuracy is a function of the inverse of heritability (Fig. 4c). However, the variability in prediction accuracy increased as the number of SNPs was reduced.

**Figure 5:**
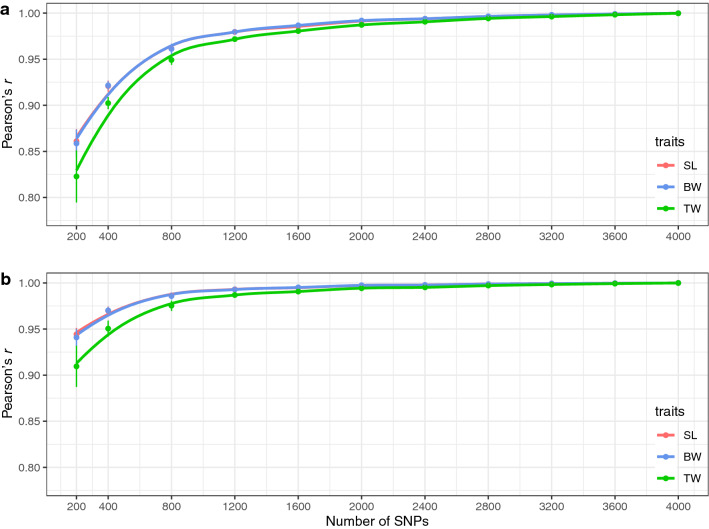
Correlations (Pearson’s *r*) of GEBVs. (**a**) correlations between GEBV estimated with the full SNP dataset (4075 SNPs) and those estimated with each SNP subset, and (**b**) correlations between the family mean GEBV estimated with the full SNP dataset and with each SNP subset for standard length (SL), body weight (BW), and testes weight (TW). Each SNP subset was sampled with ten replicates, and the mean and the standard deviation among the replicates are plotted. Loess regression lines (span = 0.5) are shown.

To assess the effect of a reduction in the number of SNPs on GEBV estimation, we also calculated the correlation coefficient (Pearson’s *r*) between the GEBV estimated from the full SNP set and those estimated from each of the 11 subsets. As for heritability and predictive ability estimation, the value of the correlation coefficient for each trait dropped when fewer than 1200 SNPs were used (Fig. 5a and Supplementary Table S4). On the other hand, when the family means of GEBVs were compared, a strong correlation (*r* > 0.95) was observed for each trait even between the full SNP set and 400 SNPs, with which family structure could be captured (Fig. 5b).

**Figure 6:**
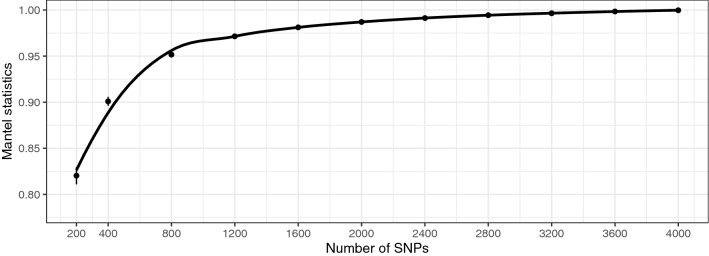
Correlations (Mantel statistics) between G matrices constructed with the full SNP set and with subsets of SNPs (200, 400, 800, 1200, 1600, 2000, 2400, 2800, 3200, 3600, and 4000 SNPs). Each SNP subset was sampled with ten replicates, and the mean and the standard deviation among the replicates are plotted. Loess regression line (span = 0. 5) is shown.

We also calculated the correlation between the realized relationship matrix (G matrix) constructed with the full SNP set and with each replicate of the 11 SNP subsets by means of the Mantel test (Fig. 6 and Supplementary Table S5). The Mantel statistics decreased gradually as the number of SNPs was reduced and a rapid decrease was observed when fewer than 1200 SNPs were used.

## Discussion

In this study, we tested the feasibility of GP for TW SL, and BW in cultured tiger pufferfish at harvest using the GBLUP model and examined the effect of varying the number of SNPs on the estimations. Moderate heritability values were obtained for the three traits (0.538‒0.686) (Fig. 3), within the range (but higher in the case of gonad weight) of those estimated in previous studies where genomic information was used, e.g. Atlantic salmon (*Salmo salar*; length: 0.61, weight: 0.60)^29^, common carp (*Cyprinus carpio*; length: 0.33)^30^, Nile tilapia (*Oreochromis niloticus*; weight: 0.36)^31^, channel catfish (*Ictalurus punctatus*; weight: 0.34)^32^, large yellow croaker (*Larimichthys crocea*; length: 0.59, weight: 0.60, gonad weight: 0.37)^33,34^, yellowtail kingfish (*Seriola lalandi*; length: 0.43, weight: 0.47)^35^, and the tiger pufferfish (length: 0.41)^22^. Prediction accuracy was relatively high for all of the traits (prediction accuracy: 0.665‒0.792), but also within the range of previous studies: Atlantic salmon (weight: 0.70, length: 0.66)^29^, common carp (length: 0.71)^30^, Nile tilapia (weight: 0.60)^31^, channel catfish (weight: 0.37)^32^, large yellow croaker (length: 0.40, weight: 0.41)^33^, yellowtail kingfish (length: 0.67, weight: 0.69)^35^, and the tiger pufferfish (length: 0.73)^22^. The high heritability and prediction accuracy indicate that GS can be applied for selective breeding on TW in addition to SL and BW of the tiger pufferfish.

The predictive ability and the prediction accuracy estimated under the GBLUP model with 4075 SNPs was higher than those estimated under the inf-PBLUP model for each trait (Fig. 3). This indicates that the medium density SNP set could successfully capture not only the family structure but also the genetic variation among full-sibs due to Mendelian sampling. Indeed, the parent–offspring pair was uniquely assigned, and parents were clearly distinguished from each other with the 4075 SNP set even though they were made up of full- and half-sibs. Family structure analysis showed that even a smaller number of SNPs (400 SNPs) could capture the family structure of the population. However, this number of SNPs was not sufficient for accurate estimation of the additive genetic relationship between individuals as the correlation of the G matrix was low (Fig. 6). Judging from the Mantel statistics, more than 1200 SNPs are required to handle the genetic variation between full sibs arising due to Mendelian sampling, as a rapid reduction in the statistics was observed with fewer SNPs. Similarly, rapid changes in the estimated heritability, predictive ability, and prediction accuracy were observed with SNP subsets fewer than 1200 SNPs for each trait (Fig. 4). These results also support the observation that more than 1200 SNPs are needed to handle both family structure and the Mendelian sampling error simultaneously, and thus to attempt GP for the tested population. In this study, heritability estimated under inf-PBLUP model was higher than those estimated under GBLUP. This is most likely due to an over-estimation of the additive genetic variances under PBLUP since PBLUP is less powerful for separating genetic and non-genetic effects compared to GBLUP^36^.

To investigate further the effects of reducing the number of SNPs on the prediction performance, we calculated the correlation coefficient (Pearson’s *r*) between the GEBVs estimated with the full SNP dataset and with each of the SNP subsets (Fig. 5a). As in the case of the genetic statistics mentioned above, a drop in the value of the correlation coefficients was observed in each trait when fewer than 1200 SNPs were used. On the other hand, the family mean of GEBVs estimated with 400 SNPs showed a higher correlation with that estimated with the full SNP set (*r* > 0.95) (Fig. 5b). These results also indicate that the genetic variation derived from the differences among families could be captured with 400 SNPs, but that genetic variation due to differences among full siblings was not. Taken together, it can be concluded that GP for SL, BW, and TW is possible for the cultured population of the tiger pufferfish using a medium density of SNPs, i.e., more than 1200 SNPs.

Kriaridou et al.^14^ reported that a medium number of SNPs of 1000–2000 would be sufficient to gain a prediction accuracy as high as those obtained from high-density panels (7–10 K SNPs), regardless of the species, population and family structure, traits, and genotyping platforms. This is particularly the case when the training population used for the prediction model construction and the selection candidates are closely related, because long genomic segments which can be captured with a small number of SNPs are shared among individuals. Our results also suggest that a medium number of SNPs (2–4 K) is sufficient for GS programs in the tiger pufferfish population. The prediction performance of the GBLUP model is superior to that of the BLUP model mainly due to the improvement in estimation of genetic relationship between each pair of individuals. Our results revealed that a larger number of SNPs are required for accurate estimation of genetic relatedness compared to estimation of family structure. This suggests that the SNPs informative for the estimation of genetic relatedness among individuals and for the inference of family structure are different. Therefore, SNP selection based on, for example, site *F*_*ST*_, principal components analysis (PCA), and random forest, which account for the effects of each SNP on the family structure, would not help increase the prediction performance, especially for polygenic traits. To further decrease the number of SNPs for cost effective prediction, genotyping imputation from using a low to medium density panel^37^ would be a better choice compared to non-random SNP selection schemes.

In conclusion, our data revealed that GS is available for improvement of TW as is the case for SL and BW in selective breeding of the tiger pufferfish^22^. A medium size dataset of SNPs (4075 SNPs) is sufficient for accurate prediction, but the number of SNPs can be reduced to 1200 without much loss of accuracy. Below this threshold, however, genetic relationship between each pair of individuals is likely to be obscured and the prediction accuracy will deteriorate.

## Methods

### Specimens

We purchased 996 individuals (F_0_ generation) from aquaculture farms in Nagasaki prefecture (Japan) in December 2016. Some of these fish (*n* = 652) were used in a previous study^26^, and the remaining (*n* = 344) were analyzed specifically for this study. Approximately half of the entire group of fish (*n* = 495) was transferred to Nagasaki Prefectural Institute of Fisheries (NPIF, Nagasaki, Japan) as the broodstock candidates without phenotype recording, while the rest of fish were sacrificed for phenotyping at site. A fin-clip was obtained from each individual for genotyping, stored in 99.5% ethanol and kept at − 30 °C until DNA extraction. In April 2018, nine females and nineteen males were crossed to produce the F_1_ generation (total of 129 full-sib families). The genetic relationships among the parents were assessed as explained below prior to the mating to minimize inbreeding between full sibs. The F_1_ generation was raised at NPIF in the conventional manner^17,25^. At 20 months old, 712 individuals were sampled; 500 (male = 246, female = 254) were euthanized with an overdoze of 2-phenoxy ethanol (> 600 mL/ton) and body size data (SL and BW) was collected. Testes weights was also measured for each male. A fin-clip was collected from each fish for genotyping. The remaining 212 fish were retained as broodstock. The sample information is presented in Supplementary Tables S6 and S7. The histogram of each trait (Fig. 1) was created using R/ggplot2 (v3.3.5)^38^. All the other figures were also created using R/ggplot2 (v3.3.5) with the exception of Supplementary Fig. S1, drawn using Microsoft PowerPoint 2016.

### Genotyping

Genotyping of genome-wide SNPs was performed using the Ampliseq custom panel (Thermo Fisher Scientific Inc) as described previously^26^. In brief, genomic DNA was extracted from the fin-clip and used for genotyping. The first PCR was carried out using custom AmpliSeq primer pools that include 3187 target loci. P7/P5 Illumina adaptors and custom-designed 8 bp dual indices were then added to a second PCR. For the F_0_ generation, we produced four library pools with 326, 326, 305 and 39 samples, respectively. The two library pools with 326 samples were used in the previous study. For the F_1_ generation in the current study, three library pools of 232, 267, and 213 individuals were constructed (total 712 individuals). Paired-end sequencing was carried out using an Illumina MiSeq with a MiSeq Reagent Kit (v2, 300 cycles, Illumina), or a MiSeq Reagent Micro Kit (for the library consisting of 39 samples). Quality-trimming was performed using Trimmomatic (v0.38)^39^ with the following filtering parameters: *ILLUMINACLIP TruSeq2-PE-2.fa:2:30:10, SLIDINGWINDOW:30:20, AVGQUAL:20*. The read pairs surviving at both ends were mapped onto the reference sequence covering 50 bp upstream and downstream of the targeted loci extracted from FUGU5/fr3^16^ using BWA-mem^40^. The file format was converted from SAM to BAM using SAMtools^41^ and used for SNP genotyping. GATK HaplotypeCaller (v4.1.4)^42^ was used to detect polymorphisms with following parameters: *–output-mode EMIT_ALL_CONFIDENT_SITE, –stand-call-conf 30* and *–ERC BP_resolution*. Genotyping of each sample was performed using GATK GenotypeGVCFs with *–all-site TRUE* option. This per-base genotyping allows us to distinguish between the reference homozygote and the missing genotype. Low quality SNPs were filtered out using VCFtools (v0.1.17)^43^ with options of *–minDP 5* and *–minQ 30*. Genotype files were merged per generation using BCFtools *merge* (v1.9)^44^, and meanwhile, only SNPs with two alleles, minor allele frequency (MAF) larger than 0.01 and MAF less than 0.4 were selected for each generation using BCFtools *view*. Subsequently, SNPs with genotyping rate less than 60% were removed from the F_0_ generation using VCFtools. The remaining SNPs were then extracted from the F_1_ generation and SNPs with genotyping rate less than 60% were excluded. As a result, 4075 high quality SNPs surviving in both generations were retained.

The effect of the number of SNPs was examined after genotype imputation. The imputation step was performed for each generation using LinkImpute (v1.1.4)^45^ with default setting. The imputation strategy adopted in the software depends on LD-kNNi (linkage disequilibrium, *k*-nearest neighbors imputation), which is free from pedigree information. Subsequently, subsets of SNPs (200, 400, 800, 1200, 1600, 2000, 2400, 2800, 3200, 3600, and 4000) were randomly extracted from the imputed SNP panel using the bash *shuf* command. Ten replicates were produced for each subset independently. Genotype vcf files were converted into ped, bed or 012 (recodeA) format using VCFtools and PLINK v1.07^46^ as needed.

### Pedigree reconstruction

As no pedigree information was available for the original aquaculture population, we have reconstructed the pedigree from the pre-imputed data set (4075 SNPs). Pair-wise kinship coefficients were estimated among the 28 parental individuals using KING software^27^ to infer kin relationships among them based on the coefficient range (1st-degree: 0.177–0.354; 2nd-degree: 0.0884–0.177; 3rd-degree: 0.0442–0.0884) following the manual. Meanwhile, parentage assignment was carried out using ADMIXTURE v1.3.0^28^. Optimal *K*-value (the number of subpopulations) was selected as 26–42 based on five-fold cross validation implemented in the software. Parentage assignment was also carried out using one of the ten replications of the imputed SNP subsets (200, 400, and 800 SNPs). Finally, the pedigree was reconstructed from the results of kin relationship among parents and parentage.

### Heritability estimation

Narrow-sense heritability was estimated for SL, BW, and TW in the F_1_ generation with phenotypic data from 695 individuals for SL and BW, and 246 males for TW, using rrBLUP package^47^ for the 11 subsets of imputed SNPs (including ten replicates). The following genomic BLUP (GBLUP) model was fitted:

$${\text{y}} = \mu + {\text{Za}} + {\text{e}},$$

where y and μ are the vectors of the observed phenotypes and phenotypic means, respectively; a and e are vectors of the additive genetic effects and residuals, respectively; and Z is the corresponding incidence matrices for additive effects. The additive genetic effects follow the normal distribution ~ N(0, Gσ_a_^2^), where G is the genomic relationship matrix, calculated using *A.mat* function, and σ_a_^2^ is the additive genetic variance. Narrow sense heritability was calculated as *h*^2^ = σ_a_^2^/(σ_a_^2^ + σ_e_^2^).

Narrow-sense heritability of each trait was also estimated based on the inferred pedigree information (inf-PBLUP). The same model as GBLUP was fitted, but the G matrix was replaced by the numerator relationship matrix (A); the additive genetic effects follows the normal distribution ~ N(0, Aσ_a_^2^). The A matrix was calculated using *Amatrix* function of AGHmatrix^48^, and the model was solved using rrBLUP.

### Breeding value prediction

The GEBV and inf-EBV of each trait of each fish were predicted under the linear model described above. The estimation of GEBV was also performed using the ten replicates of the 11 SNP subsets. The predictive ability and the prediction accuracy were estimated for each dataset using ten replicates of tenfold cross-validation as described in Hosoya et al.^49^. In brief, individuals were randomly split into ten groups, and nine were used as the training set to predict GEBV of the reminder (validation set) of which phenotype recodes were masked. Predictive ability was determined as the correlation between the observed phenotype and predicted GEBV of the validation set. Prediction accuracy was obtained by dividing predictive ability by the square root of the heritability estimated above. This set was repeated ten times while rotating the validation set among the ten groups, and the whole procedure was repeated ten times independently to obtain the mean and the standard error of the measure of prediction accuracy.

Pearson’s correlation coefficients (*r*) were calculated among GEBVs and among the family means of GEBVs calculated with the full SNP set and each replicate SNPs subset. The correlation among the G matrices averaged over the replicates and A matrix was also calculated as the Mantel statistics using the *mantel* function implemented in vegan package^50^.

### Ethics statement

All the experiments were approved by the local Fish Care Committee of Nagasaki Prefectural Institute of Fisheries (NPIF) (#NPIF-0001) and carried out in accordance with the Guidelines for Fish Experimentation in NPIF. This study was carried out in compliance with the ARRIVE guidelines for fishes.

## Supplementary Information

Supplementary Figures.

Supplementary Tables.

## Data Availability

Amplicon sequence reads have been deposited in the DDBJ Sequence Read Archive (Submission: DRA007457–DRA007464, DRA011515). Accession number for each individual is listed in Supplementary Tables S5 and S6. The phenotype of each individual (if collected) is also listed in the Supplementary Tables.
