# Supplementary material for: Genomic prediction for testes weight of the tiger pufferfish, Takifugu rubripes, using medium to low density SNPs
Source: Sci Rep. 2021 Oct 13;11:20372. doi: 10.1038/s41598-021-99829-1 (PMC8514491; doi:10.1038/s41598-021-99829-1)
Supplement: Supplementary file 1 — Supplementary Figures. [file 41598_2021_99829_MOESM1_ESM.docx]

**Supplementary Material**

Article title: Genomic prediction for testes weight of the tiger pufferfish, *Takifugu rubripes*, using medium to low density SNPs

Authors: Sho Hosoya^1^, Sota Yoshikawa^2^, Mana Sato^1^, Kiyoshi Kikuchi^1^

^1^Fisheries Laboratory, University of Tokyo, Hamamatsu, 431-0214, Japan

^2^Nagasaki Prefectural Institute of Fisheries, Nagasaki, Japan

*Corresponding author

Sho Hosoya

e-mail: ahosoya@mail.ecc.u-tokyo.ac.jp

TEL: +81-53-592-2821; FAX: +81-53-592-2822

Supplementary Fig. S1. Estimated kin relationship among the 28 parental individuals.

Supplementary Fig. S2. *K* value selection in the genetic admixture analysis.


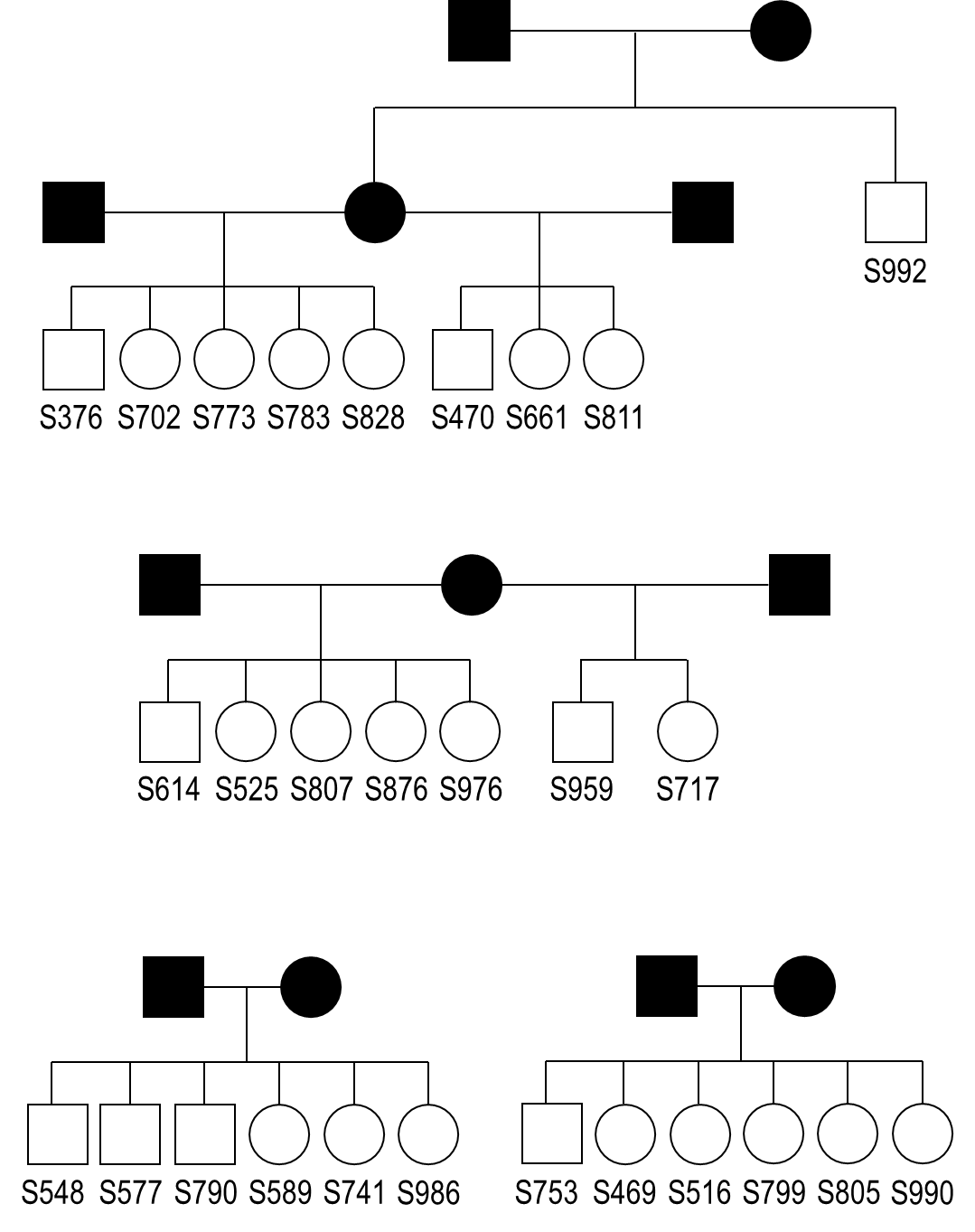


Supplementary Fig. S1.

Estimated kin relationship among the 28 parental individuals. Open squares and circles indicate females and males used in this study. The ID of each individual is shown below the square/circle. Black squares and circles are inferred ancestral individuals not used in this study.


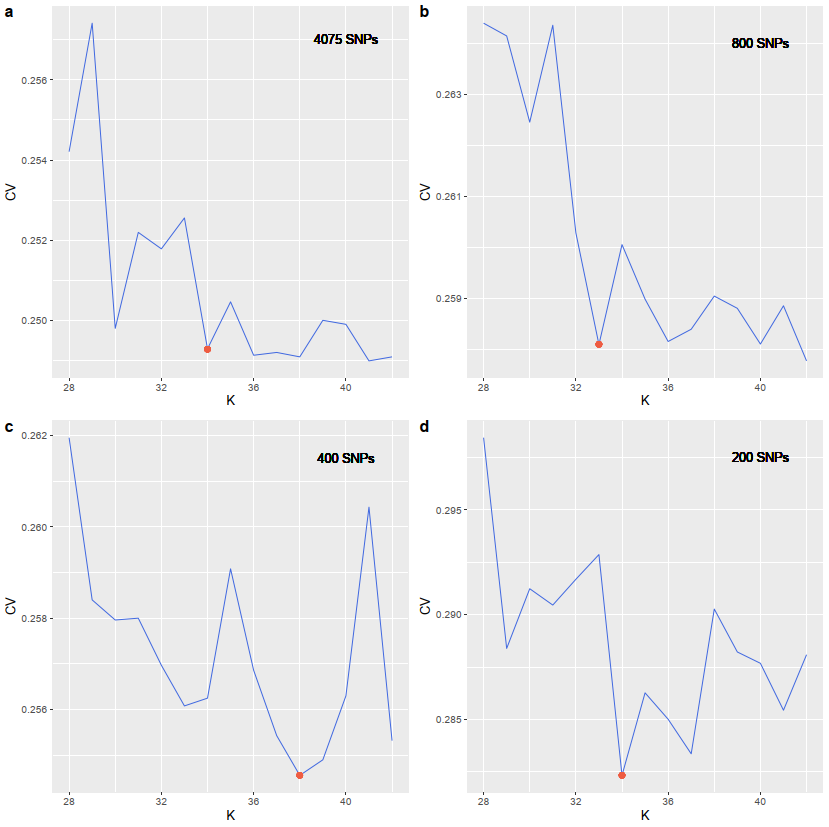


Supplementary Fig. S2.

*K* value selection in the genetic admixture analysis. Error rates estimated by cross validation (Y-axis) for each K value (28 to 42, X-axis) are plotted for the full SNP set (a), 800 SNPs (b), 400 SNPs (c), and 200 SNPs (d). Red circles indicate the selected *K* value.
